# Insights into the impact of hepatitis B virus on hepatic stellate cell activation

**DOI:** 10.1186/s12964-023-01091-7

**Published:** 2023-04-11

**Authors:** Hongjuan You, Xing Wang, Lihong Ma, Fulong Zhang, Huanyang Zhang, Yuxin Wang, Xiucheng Pan, Kuiyang Zheng, Fanyun Kong, Renxian Tang

**Affiliations:** 1 Jiangsu Key Laboratory of Immunity and Metabolism, Jiangsu International Laboratory of Immunity and Metabolism, Department of Pathogenic Biology and Immunology, Xuzhou Medical University, Xuzhou, Jiangsu China; 2 Imaging Department, The Second Affiliated Hospital of Shandong First Medical University, Taian, China; 3 Department of Infectious Diseases, The Affiliated Hospital of Xuzhou Medical University, Xuzhou, China; 4 National Demonstration Center for Experimental Basic Medical Sciences Education, Xuzhou Medical University, Xuzhou, Jiangsu China

**Keywords:** Liver fibrosis, Hepatitis B virus, Inflammation, Hepatic stellate cells

## Abstract

During chronic hepatitis B virus (HBV) infection, hepatic fibrosis is a serious pathological condition caused by virus-induced liver damage. The activation of hepatic stellate cells (HSCs) is a central event in the occurrence and progression of liver fibrosis. Although accumulating evidence has shown that HBV directly stimulates HSC activation, whether the virus infects and replicates in HSCs remains controversial. Inflammation is one of the obvious characteristics of chronic HBV infection, and it has been demonstrated that persistent inflammation has a predominant role in triggering and maintaining liver fibrosis. In particular, the regulation of HSC activation by HBV-related hepatocytes via various inflammatory modulators, including TGF-β and CTGF, in a paracrine manner has been reported. In addition to these inflammation-related molecules, several inflammatory cells are essential for the progression of HBV-associated liver fibrosis. Monocytes, macrophages, Th17 cells, NK cells, as well as NKT cells, participate in the modulation of HBV-related liver fibrosis by interacting with HSCs. This review summarizes current findings on the effects of HBV and the relevant molecular mechanisms involved in HSC activation. Because HSC activation is essential for liver fibrosis, targeting HSCs is an attractive therapeutic strategy to prevent and reverse hepatic fibrosis induced by HBV infection.

**Video abstract**

**Supplementary Information:**

The online version contains supplementary material available at 10.1186/s12964-023-01091-7.

## Introduction

Although effective hepatitis B virus (HBV) vaccines have been applied in most countries, chronic infection of the virus remains a global health threat affecting nearly 300 million people. Available therapies, including nucleos(t)ide analogs and interferons, restrict HBV replication. However, these antiviral treatments cannot remove HBV from hepatocytes to cure HBV-related diseases, including inflammation, fibrosis, cirrhosis, and liver cancer, owing to the stable existence of covalently closed circular deoxyribonucleic acid (cccDNA) of the virus in host cells, integration of the viral genome into the host chromosome, and incapacity of the host´s immune response to eliminate the virus [1]. The viral genome contains S, C, P, and X overlapping open reading frames. ORF S is responsible for the expression of preS1-Ag, preS2-Ag, and HBsAg. ORF C contributes to the production of a capsid protein HBcAg and a secreted viral protein HBeAg. ORF P and X encode two nonstructural viral proteins: HBV polymerase and HBX protein [2]. Accumulating data has indicated that HBV particles and viral antigens have developed various strategies to utilize host factors and machinery to establish and maintain HBV infection [3, 4], leading to the occurrence and progression of various liver disorders [5]. Therefore, to better identify potential targets for treating virus-relevant diseases, a deeper understanding of the mechanisms responsible for HBV-induced illnesses is urgent.

Liver fibrosis, a wound-healing response to persistent hepatic injury induced by the infection of HBV, can lead to cirrhosis, liver cancer, and eventually, death [6]. Although available clinical data show that anti-HBV therapy has the capability of suppressing the progression of liver fibrosis [7], a direct approved anti-fibrotic treatment is unavailable [8]. Therefore, a better comprehension of the mechanisms that contribute to fibrosis regulated by HBV is required to elucidate how this disorder can be alleviated. Hepatic stellate cell (HSC) activation is a central event in liver fibrosis [9, 10]. Although recent investigations have suggested that direct activation of HSCs mediated by HBV is associated with liver fibrosis [11], whether HBV can directly infect HSCs has not been fully confirmed. After HBV-induced liver injury, hepatocytes release a variety of soluble inflammation-related molecules, such as TGF-β and CTGF [12, 13], to accelerate HSC activation. A variety of inflammatory cells, including macrophages [14], T cells [15], as well as NK cells [16], are also responsible for the regulation of HSC activation with the production of extracellular matrix (ECM) proteins, including collagen I (COL1A1) and α-SMA. Excessive ECM accumulation destroys the normal liver architecture, leading to fibrosis and cirrhosis. Here, we provide a comprehensive overview of the underlying mechanisms by which HBV stimulates HSC activation to modulate liver fibrosis.

### Direct activation of HSCs stimulated by HBV

It has been reported that HBV particles from HepG2.2.15 cells can transiently infect HSCs, specifically LX-2 cells, leading to HBsAg and HBcAg production in the cytoplasm of virus-infected HSCs and affecting their proliferation and expression of COL1A1 [11]. The viral particles derived from HepG2.2.15 cells also upregulate the expression of α-SMA, PDGF-B, and PDGFR-β, as well as enhance the phosphorylation of PDGFR-β, in HSCs [17]. In addition, HepG2.2.15 cell-secreted HBV represses c-Jun/AP-1 activation in LX-2 cells, leading to the attenuation of apoptosis in HSCs [18]. Nevertheless, Wu et al. found that purified HBV particles from chronic hepatitis B (CHB) patients could not infect LX-2 cells but could directly increase COL1A1 expression in HSCs outside the cells. Wu et al. concluded that HBsAg might be implicated in the expression of COL1A1 in HSCs stimulated by extracellular HBV particles [19], whereas Liu et al. showed that HBsAg has no direct effect on HSC activation [20]. To date, the reasons for these discrepancies remain unaddressed, and studies are required to investigate whether HBV can directly infect and replicate in HSCs to facilitate their activation and/or whether extracellular HBV particles are capable of stimulating HSC activation.

As the current reports have provided evidence linked to the infection of HSCs by HBV in vitro, HBV-infected HSC models have been established to explore the virus on the regulation of HSC. Using cell models, it was found that based on CXCR3 [21], HBV accelerates growth and migration, but suppresses apoptosis, of HSCs via the Toll-like receptor (TLR)/Myd88 pathway. HBV induces tripartite motif-containing protein 37 (TRIM37) expression in HSCs. TRIM37 interacts with Smad7 and subsequently enhances Smad7 degradation to sensitize HBV-infected HSCs. Furthermore, aberrant activation of the NF-kB pathway controlled by reactive oxygen species contributes to the overexpression of TRIM37 by HBV [22]. Similar to TRIM37, HBV enhances the expression of TRIM52 in HSC. TRIM52 represses protein phosphatase magnesium 1A (PPM1A) and activates the Smad2/3 pathway to promote HSC activation [23].

Evidence from HBX-transgenic mice indicates that the viral molecule has a prominent effect on the progression of HBV-associated hepatic fibrosis [24]. HBX-transfected HSCs were constructed to examine the effect of HBX on liver fibrosis. Based on HBX-related HSC models, it has been shown that the viral protein can bind to PPARγ and repress its levels to maintain HSC activation [25]. Kuo et al. have shown that the viral protein induces HSC activation via the inhibition of ferroptosis-dependent pathways [26]. In addition to HBX, in HBeAg-transfected HSCs, the viral molecule has the capability of inducing TGF-β expression [27]. TGF-β, in turn, stimulates the growth of HSCs and results in the production of ECM-associated factors, including TIMP-1, α-SMA, and COL1A1.

### Paracrine activation of HSC by HBV-infected hepatocytes through inflammation-related factors

**Fig. 1:**
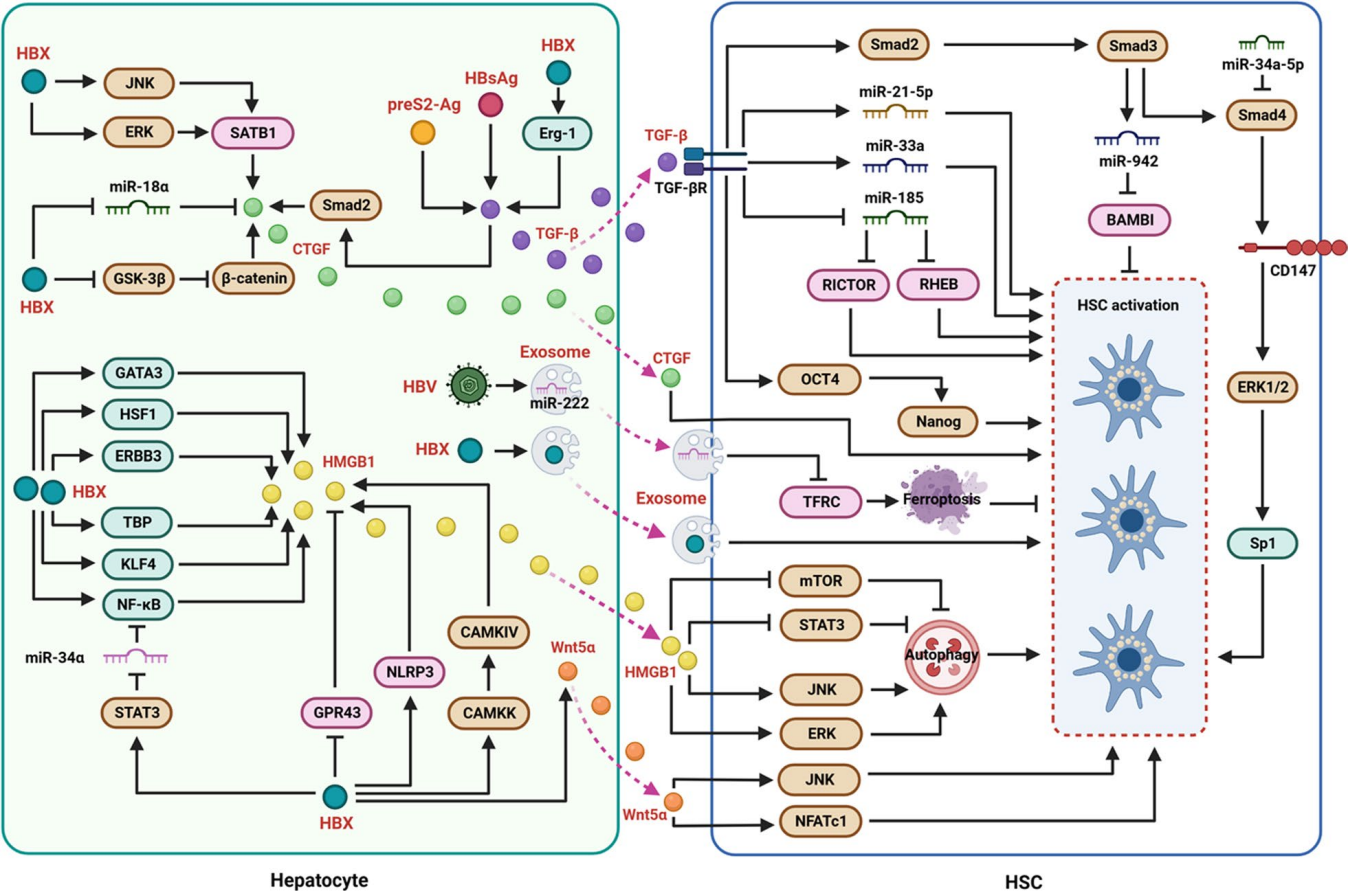
Molecular mechanisms related to the soluble molecules secreted (mediated by HBV) from hepatocytes and viral antigens that activate HSCs in a paracrine manner. In hepatocytes, HBX enhances TGF-β expression through Erg-1. In addition, HBsAg and preS2-Ag are involved in upregulation of TGF-β expression. HBX activates JNK and ERK to increase SATB1 expression levels, inhibits GSK-3β, sensitizes β-catenin, and activates the TGF-β/Smad2 pathway to upregulate CTGF expression. HBX increases HMGB1 expression levels, via activation of transcription factors GATA3, HSF1, ERBB3, TBP, KLF4, and NF-κB. The activation of STAT3 to restrict miR-34α and then activate NF-κB, the inhibition of GPR43, the activation of NLRP3, and the activation of CAMKK/CAMKIV pathway contributes to the increase of HMGB1 expression mediated by HBX. Depending on its interaction with TGF-βR, TGF-β activates the Smad2/3/4 pathway to enhance CD147 levels. CD147 activates HSCs by activating the ERK1/2 pathway and transcription factor Sp1. TGF-β upregulates miR-21-5p, miR-33a, and miR-942 to activate HSCs. miR-34α-5p can inhibit Smad4 to reduce HSC activation. Inhibition of miR-185 mediated by TGF-β promotes RICTOR and RHEB expression to sensitize HSCs. The OCT4/Nanog pathway also participates in TGF-β-mediated activation of HSCs. HMGB1 inhibits the mTOR and STAT3 pathways, but activates the JNK and ERK pathways, to induce autophagy and then activates HSCs. Additionally, the HBX mutation enhances Wnt5α expression to enhance HSC activation by activating the JNK and NFATc1 pathways. HBV promotes exosomes containing miR-222 and sensitizes HSCs by inducing TFRC-associated ferroptosis. Exosomes with HBX also activate HSCs

It has been demonstrated that chronic liver inflammation can drive hepatic fibrosis [28, 29]. In the liver tissue chronically injured by HBV, hepatocytes secrete a variety of cytokines, chemokines, and soluble inflammatory modulators, including TGF-β and PDGF, to induce HSC activation. Exosomes, which are secreted from HBV-infected cells, carry microRNAs (miRNAs), proteins, and other components that facilitate the transduction of cellular information [30, 31] and are involved in inflammatory responses [32]. In particular, exosomes from HBV-infected hepatocytes contained HBX and specific miRNAs that can be transported into HSCs to sensitize them (Fig. 1). Here, we summarized the effects of soluble inflammation-related factors and the underlying molecular mechanisms on the regulation of liver fibrosis induced by HBV.

### Role of TGF-β in HSC activation mediated by HBV-infected hepatocytes

TGF-β is a vital regulator of inflammation and fibrosis [33] and participates in the modulation of various biological processes mediated by HBV [34]. Particularly, the cytokine regulates the progression of liver inflammation and fibrosis [35, 36]. During HBV infection, HBX can induce the production and secretion of the cytokine in liver cells to sensitize HSCs with increased levels of COL1A1 and CTGF [13, 37]. TGF-β was transcriptionally activated in HBX-transfected hepatoma cells and HBX-transgenic mice. HBX-mediated TGF-β transcription is mainly mediated by the transcription factor Egr-1 [38]. HSC activation stimulated by HBX-positive hepatocytes can be prevented by neutralizing monoclonal antibodies targeting TGF-β [13]. In addition to HBX, preS2-Ag and HBsAg have also been found to stimulate TGF-β secretion from hepatocytes to activate HSCs [20, 39], however, potential regulatory mechanisms have not been reported. The results of whole-genome expression arrays showed that ITGBL1 expression was upregulated in liver biopsy samples from CHB patients having liver fibrosis. In vitro assays indicated that ITGBL1 participates in the modulation of HSC activation by upregulating the expression of TGF-β in hepatocytes [40]. However, the mechanism underlying HBV-mediated ITGBL1 expression remains unclear.

During HBV infection, HSC activation modulated by TGF-β, which is secreted by HBX-positive hepatocytes, relies on the interaction of TGF-β with its receptor (TGF-βR). TGF-β activates TGF-βR, sensitizes the Smad2/3/4 pathway, and upregulates the transmembrane protein CD147, which further enhances the levels of α-SMA and COL1A1 in HSCs by sensitizing the ERK1/2 pathway and the transcription factor Sp1. Further investigation indicated that CD147 is a potential target for antibody-induced HSC apoptosis to treat liver fibrosis [41]. In addition, HBX-stimulated TGF-β activates the OCT4/Nanog pathway in HSCs (Fig. 1). Activated OCT4/Nanog facilitates the expression of COL1A1, α-SMA, as well as TIMP-1, to promote HSC activation [39].miRNAs modulate various biological processes during HBV infection. To date, multiple miRNAs, such as miR‑2861 and miR‑3656, have been identified to participate in liver fibrosis with HBV infection, and some of these miRNAs are related to the TGF-β/Smad pathway [42]. Tao et al. showed that the levels of miR-942 were enhanced in liver specimens of patients with HBV-related fibrosis. Dependent on Smad2/3, TGF-β increases the expression of miR-942 in HSCs. miR-942 further degrades activin membrane-bound inhibitor (BAMBI) mRNA to facilitate HSC activation [43]. miR-21‑5p and miR-33a, which are upregulated in HBV-related fibrosis, are also involved in TGF-β mediated HSC activation [44, 45]. miR-185 expression is reduced in HBV-associated liver fibrosis. Inhibition of miR-185 mediated by TGF-β was observed to facilitate the increase in levels of RICTOR and RHEB and the subsequent activation of HSCs [46] (Fig. 1). The expression levels of miR-34a-5p also declined in CHB patients with liver fibrosis. Functional assays have shown that miR-34a-5p can suppress hepatic fibrosis by targeting the Smad4 to restrain the TGF-β pathway in HSCs [47].

### Effect of CTGF in HSC activation mediated by HBV-infected hepatocytes

CTGF is a secreted matricellular protein essential for the development of liver fibrosis [48]. The expression levels of CTGF in healthy livers were very low. Nevertheless, clinical observations have shown that CTGF expression in the sera and fibrotic liver tissues of CHB patients is upregulated. A strong correlation between CTGF with liver fibrosis has also been reported [49]. HBX can induce CTGF expression and secretion in hepatocytes to stimulate HSC activation [12]. Multiple regulatory mechanisms contribute to the up-regulation of HBX-stimulated CTGF. First, HBX promotes TGF-β/Smad2 pathway activation to facilitate CTGF expression [50]. Second, HBX reduces the levels of miR-18α to elevate CTGF levels [51]. In addition, HBX stimulates the expression of SATB1 via the ERK and JNK pathways to facilitate the activation and proliferation of HSCs (Fig. 1) with increased secretion of CTGF [12]. Chen et al. showed that the HBX combination (combo) mutant increases β-catenin levels and stabilizes it via repression of GSK-3β, leading to upregulation of CTGF [52]. Although CTGF contributes to the activation of HSCs mediated by HBV infection, the cellular factors responsible for the modulation of HSCs stimulated by CTGF during HBV infection have not been identified.

### Function of HMGB1 in HSC activation mediated by HBV-infected hepatocytes

HMGB1 is a constituent of the HMG family with the functions of regulating cell death, the cell cycle, and DNA replication [53]. Particularly, extracellular HMGB1 mainly acts as a pathogen-associated molecular pattern (DAMP) molecule and activates target cells via different pattern recognition receptors not limited to TLRs and RAGE to initiate inflammatory responses [53, 54]. The observation from Li et al. indicated that the expression of HMGB1 was significantly higher in CHB patients with hepatic fibrosis than in healthy subjects [55, 56]. In vitro experiments showed that extracellular HMGB1 induced cellular autophagy by sensitizing the ERK/JNK pathways and restricting the mTOR and STAT3 pathways to activate HSCs [55] (Fig. 1).

Furthermore, current reports show that HBX is responsible for the virus-mediated production and secretion of HMGB1 [57], and a variety of host factors are involved in this biological process. First, HBX-induced HMGB1 expression in hepatocytes was noted to be associated with multiple transcription factors, including GATA3, HSF1, ERBB3, TBP, NF-κB, and KLF4 [58] (Fig. 1). Second, HBX can promote STAT3 activation to restrain miR-34α expression and subsequently activate the NF-κB pathway, to facilitate upregulation of HMGB1 expression in liver cells [59]. Third, the suppression of GPR43 is involved in the HBX-mediated increase in HMGB1 expression [60]. Fourth, the activation of the NLRP3 inflammasome benefits HMGB1 production in HBX-positive hepatocytes [57]. In addition, Chen et al. discovered that the secretion of HMGB1 from hepatoma cells triggered by HBX relies on the CAMKK/CAMKIV pathway [61].

### Role of Wnt5α in HSC activation mediated by HBV-infected hepatocytes

The Wnt pathway, a well-known cell communication mechanism [62], is subdivided into canonical and non-canonical pathways. Current evidence indicates that Wnt signaling participates in the progression of inflammation and hepatic fibrosis [63, 64], and Wnt5α is elevated in HBV-related fibrosis patients [65]. Especially, Li et al. showed that Wnt5α significantly enhanced the activation of HSC by increasing the expression levels of Col1A1, α-SMA, and TIMP-1 via activating JNK and NFATc1. Notum, an inhibitor of Wnt5α, has the capability of inhibiting HSC activation induced by HBV-associated hepatocytes (HepAD38 cells) by downregulating Wnt5α-mediated noncanonical pathways [65]. Liu et al. showed that mutations in the C-terminus of HBX can elevate Wnt5α levels in hepatoma cells [66]. However, the mechanisms that contribute to the HBX-induced increase in Wnt5α are unclear. In addition, it has been demonstrated that numerous cellular factors participate in Wnt pathway activation [62], except for Wnt5. Whether other constituents of this pathway participate in the modulation of HBV-stimulated HSC activation has not been fully clarified.

### Effect of exosomes in HSC activation mediated by HBV-infected hepatocytes

Exosomes are small extracellular vesicles released from different cells that can transfer specific proteins, nucleic acids, lipids, and other molecules from donors to recipient cells. Exosome contents vary depending on cell type and physiological conditions [67]. Zhao et al. showed that HBX had a significant effect on changes in exosome protein content secreted from HBV-infected cells [68]. Exosomes derived from HBV-infected hepatocytes can enhance HSC activation. miR-222 levels increase in HBV-related exosomes. Exosomal miR-222 derived from hepatocytes with HBV infection accelerates liver fibrosis by repressing transferrin receptor (TFRC)-mediated ferroptosis [69] (Fig. 1). Yang et al. discovered that serum exosomes from CHB patients contained HBV components, including HBV DNA, viral RNA, and HBsAg [30]. In particular, Kapoor et al. observed that HBX mRNA and protein can be transported to HSC via exosomes to stimulate HSC activation [70]. Tang et al. showed that exosomes, which could be used as a delivery system carrying siRNA or antisense oligonucleotides that specifically target STAT3, suppressed liver fibrosis and improved liver function in mice [71]. The results of this study suggest that exosomes could be used as potential drug delivery vehicles for HBV-related liver fibrosis.

### HBV induces HSC activation via inflammatory cells

**Fig. 2:**
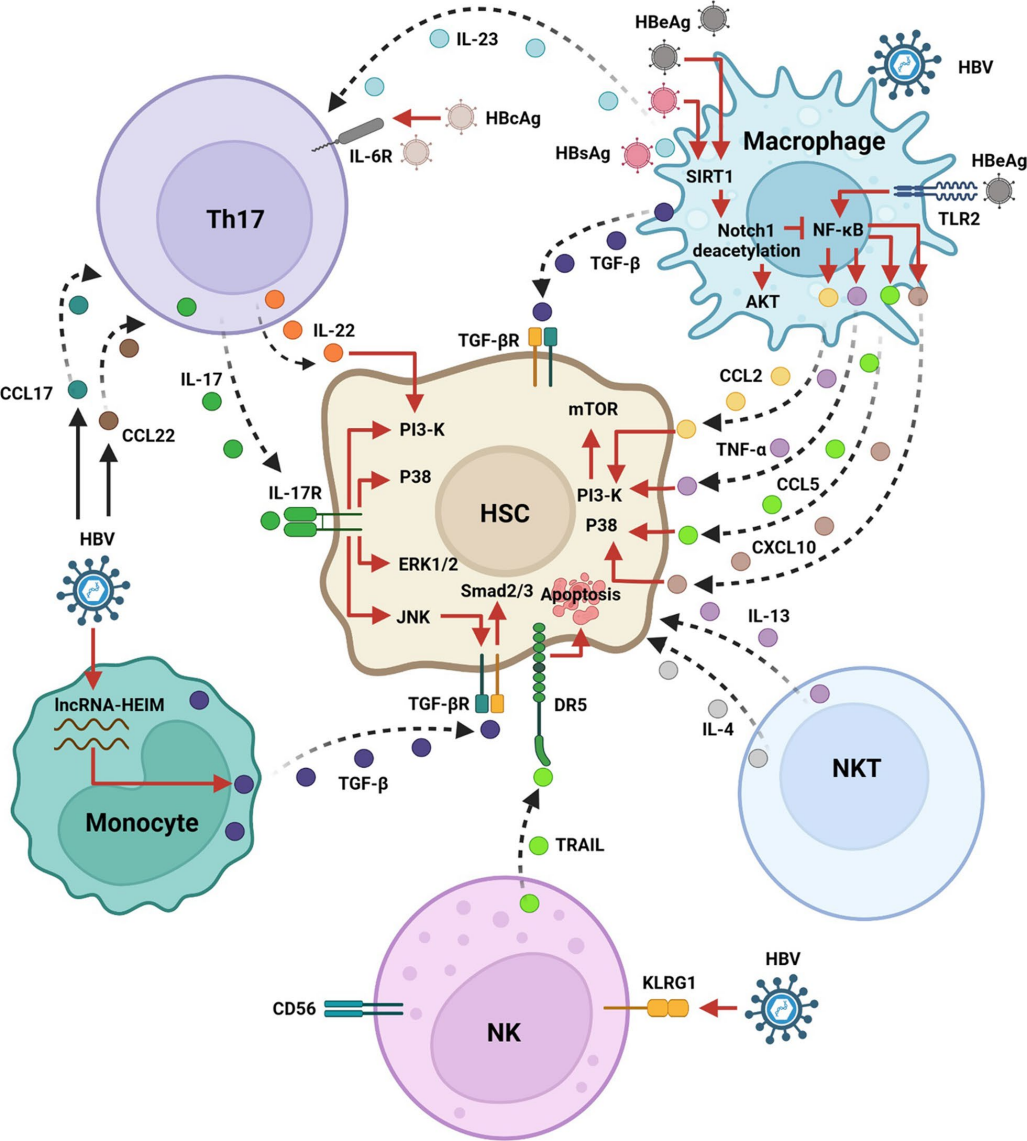
The molecular mechanisms associated with different immune cells, including monocytes, macrophages, Th17 cells, NK cells, and NKT cells, participate in the modulation of HSC activation during HBV infection. HBV promotes lncRNA-HEIM expression in monocytes to induce TGF-β activation, following which HSCs are activated. HBV stimulation can upregulate KLRG1 expression in NK cells, and KLRG1 + NK cells are capable of inducing HSC apoptosis in a TRAIL-DR5-dependent manner. During HBV infection, NKT cells activate HSCs through IL-4 and IL-13. HBV induces Th17 cell recruitment to the liver via CCL17 and CCL22. HBV activates Th17 cells via IL-23 secreted by macrophages. HBcAg stimulates Th17 cell activation via IL-6R. Activated Th17 cells secrete IL-17 and IL-22 to activate HSCs via activating PI3K. In addition, IL-17 activates HSCs by interacting with IL-17R to facilitate activation of the P38 and ERK1/2 pathways. IL-17 activates JNK and positively regulates the TGF-βR-Smad2/3 pathway. HBsAg and HBeAg promote SIRT1 expression and then induce Notch 1 deacetylation to inhibit NF-κB, but they activate AKT to induce macrophage differentiation toward M2. M2 macrophages activate HSCs by producing and secreting TGF-β. HBeAg induces macrophage activation through TLR2 and activates the NF-κB pathway to upregulate the expression and increase the secretion of CCL2, TNF-α, CCL5, and CXCL10. These cytokines and chemokines activate the PI3K-mTOR and P38 pathways in HSCs to facilitate HSC activation

During HBV infection, cell damage caused by the virus can cause the release of inflammation-related cytokines and chemokines from hepatic parenchymal cells to facilitate the recruitment and activation of various inflammatory cells, which further communicate with HSC and enhance the transformation of HSCs to induce the expansion of liver fibrosis [72]. A striking characteristic of chronic HBV infection is the aggregation and activation of monocytes, macrophages, T cells, as well as NK cells, in the liver [73, 74] (Fig. 2). These inflammatory cells play a predominant role in maintaining a local persistent inflammatory state that causes liver fibrosis via interactions between different immune cells, cytokines, and signaling pathways. Here, we discuss the effects of different inflammatory cells on HBV-mediated HSC activation.

### Effect of monocytes or macrophages in HSC activation during HBV infection

During HBV infection, the virus can cause the dysfunction of monocytes [75], which facilitates the expansion of Th17 cells in HBV-infected patients [76]. HBV can elevate levels of TGF-β produced by monocytes. In particular, the virus induces upregulation of lncRNA-HEIM in monocytes to accelerate TGF-β production and promote HSC activation (Fig. 2) [77]. However, the viral components involved in monocyte regulation to enhance HSC activation remain unknown, and additional studies are required to address this issue. In addition to monocytes, macrophages are heterogeneous in the liver. Liver macrophages not only are subdivided into monocyte-derived macrophages and tissue-resident macrophages (Kupffer cells) but also are functionally classified into two distinct phenotypes, including the classically sensitized “pro-inflammatory” M1 macrophages and alternatively activated “immunoregulatory” M2 macrophages. Recent reports have shown that HBV could enhance macrophage activation and M2 polarization [78, 79]. Subsequently, M2 macrophages facilitate liver fibrosis [80]. Li et al. showed that HBV particles can stimulate macrophage activation to produce TGF-β [81]. These cytokines may contribute to HSC activation by macrophages (Fig. 2).

Among HBV-encoded proteins, HBsAg and HBeAg facilitate the polarization of macrophage toward the M2 phenotype [82, 83]. In terms of mechanisms, HBsAg and HBeAg elevate the expression of sirtuins 1 (SIRT1), which facilitates Notch1 deacetylation, leading to the increase of Akt activation and the decline of NF-κB nuclear translocation in macrophages [82]. Xie et al. showed that based on the interaction of HBeAg with TLR2, HBeAg stimulates the activation of macrophages via NF-κB signaling and causes the secretion of CCL2, CCL5, TNF-α, and CXCL10, which may promote the growth and motility of HSC. Furthermore, the p38 MAPK and PI3K-mTOR pathways have been shown to facilitate macrophage-induced motility of HSC, and the TGF-β signaling pathway is involved in the regulation of HSC growth mediated by these cytokines and chemokines [14] (Fig. 2).

### Role of Th17 cells in HSC activation during HBV infection

T cell-mediated immunity has a vital role in controlling the infection of HBV [84]. Interleukin (IL)-17 is an important inflammatory cytokine secreted by Th17 cells. In patients with HBV infection, the expression levels of IL-17 are closely related to liver fibrosis [15, 85]. IL-17 can activate HSCs by interacting with its receptor (IL-17R) on HSCs, and subsequently, different signaling pathways participate in the activation of HSCs. For example, through the p38 and ERK1/2 signaling pathways, IL-17 can cause HSC activation to enhance liver fibrosis [86]. IL-17 can stabilize TGF-βRII in a JNK-dependent manner to induce Smad2/3 pathway activation, leading to HSC activation [87] (Fig. 2).

Circulating and intrahepatic Th17 cell frequency increases significantly in CHB patients [15]. Among viral proteins, HBsAg can induce cytokine IL-23 secretion from macrophages to facilitate naïve CD4^+^T cells differentiation toward Th17 cells [88] (Fig. 2). The virus can also accelerate the recruitment of Th17 cells to the liver via the chemokines CCL17 and CCL22 [89]. Similar to HBsAg, HBcAg stimulates Th17 cell production. IL-10 suppresses HBcAg-stimulated Th17 cell activation; however, administering a neutralizing antibody against IL-10 increases the number of Th17 cells [90]. The increase in the population of circulating Th17 cells mediated by HBcAg is also related to the frequency of CD4^+^T cells expressing the IL-6 receptor (IL-6R), and the frequency of the T cell subset can be repressed using an anti-IL-6R monoclonal antibody [91].

Current studies have shown that multiple host factors participate in the modulation of Th17 cells during HBV infections. For example, the number of liver-infiltrating IL-22+T cells increases in patients with HBV-associated liver fibrosis and is positively correlated with the liver fibrosis staging score. In HBV-transgenic mice, the blockade of IL-22 attenuates the levels of CCL20 and CXCL10 in the liver and reduces Th17 recruitment to inhibit liver fibrosis [92]. HBX plays a vital role in HSC activation. Zhang et al. indicated that HBX-mediated activation of HSCs can recruit more Th17 cells into the liver and promote the secretion of IL-17 and IL-22, which, in turn, induces HSC growth and the production of fibrotic markers. Moreover, the activation of HSCs stimulated by IL-17 and IL-22 is dependent on the PI3K/AKT pathway [93]. Conversely, the induction of Th17 cells mediated by activated HSCs relies on the inflammatory cytokines IL-1β and IL-6 and the activated COX-PGE2 pathway [94].

In addition to Th17 cells, an elevated frequency of IL-21+CD4^+^ cells and increased expression levels of IL-21 have been discovered in CHB patients with cirrhosis. The use of IL-21 in vitro can increase collagen production in HSCs [95]. A higher frequency of circulating CD161+CD4^+^T cells was also observed in CHB patients. The T cell subtype exerts pro-fibrogenic effects by modulating HSCs via the IL-23/IL-17 axis [96].

### Function of NK cells and NKT cells in HSC activation during HBV infection

In the liver, NK cells are the predominant subtype of lymphocytes [97], which play vital roles in the removal of infectious agents, and their activity is dependent on the modulation of activating or inhibitory receptors. The current study shows that in comparison with healthy subjects, the number and activity of NK cells were decreased in CHB patients and HBV-associated liver cirrhosis patients. In particular, the expression of activation receptors, including NKp46, NKG2D, and NKp44; activation markers, such as HLA-DR, TRAIL, as well as CD69; and cytolytic molecules, including granzyme A and perforin, was downregulated, while that of the inhibitory receptor CD158b was upregulated in chronic HBV-associated liver cirrhosis patients, in comparison with CHB patients [98]. In addition, although no difference was found in the frequency of NK cells between early and advanced HBV-associated fibrosis, the expression levels of perforin, IFN-γ, as well as NKp46, in intrahepatic NK cells declined in patients with CHB-associated advanced liver fibrosis [99].

Current evidence has shown that NK cells can inhibit hepatic fibrosis based on their cytotoxicity to target activated HSC [100], while the anti-fibrotic activity of the immune cells in patients with HBV-associated cirrhosis is impaired [98]. Elevated TGF-β levels were found to be responsible for the decline in the anti-fibrotic activity of NK cells in CHB-related liver cirrhosis patients. The results from Shi et al. have indicated that intrahepatic NK cells directly interact with HSCs in vivo. Depending on the TGF-β-dependent mechanism, HSCs can suppress the antifibrotic activity of NK cells. Among NK cell subtypes, the frequency of TRAIL + CD56^bright^ NK cells is elevated in HBV-associated liver cirrhosis patients and is negatively related to liver function, but positively associated with the degree of fibrosis [101]. KLRG1 is an NK cell inhibitory receptor, and increased hepatic KLRG1 + NK cells were observed in CHB patients. However, increased levels of KLRG1 + NK cells were associated with a decreased stage of fibrosis. KLRG1 + NK cells can interact with HSCs and, in a TRAIL-DR5-dependent manner, the immune cells cause HSC apoptosis [16] (Fig. 2). Wijaya et al. found that chronic HBV infection stimulates the expression of KLRG1 in NK cells; nevertheless, the underlying mechanism still unclear.

NKT cells, which have the characteristics of expressing the surface markers of both NK and T cells, are heterogeneous immunoregulatory cells abundant in the liver. The immune cells can recognize lipid antigens and perform a variety of functions in liver diseases [102]. Although current investigations have shown that NKT cells were capable of inhibiting the activation of HSCs in mice [103], increased liver NKT cells contribute to the virus-mediated activation of HSCs. In this process, cytokines IL-13 and IL-4 produced by NKT cells were found to facilitate HSC activation [74] (Fig. 2). The host factors that participate in regulating the abnormality of NKT cells in HBV-mediated HSC mediated by HBV are still unclear. In addition, invariant natural killer T cells (iNKTs) are the major subtypes of NKT cells that can recognize lipids and express invariant T cell receptor α-chains [104]. Wei et al. found that the populations of peripheral iNKT cells decreased but were activated in CHB patients with cirrhosis, and they speculated that the decrease of peripheral iNKT cells may be related to their migration from the periphery to the liver. Based on IL-13 or IL-4, iNKT cells facilitate HSC activation, and the influence of iNKT cells on HSC may be responsible for the progression of hepatic fibrosis in CHB patients [105]. However, the host factors that participate in virus-mediated modulation of iNKT cells have not been fully elucidated.

## Conclusion

During HBV infection, hepatic fibrosis is a very complicated pathological process that is triggered by liver damage caused by the virus. Various inflammatory cells, inflammation-related cytokines, chemokines, and signaling pathways participate in regulating the occurrence and progression of hepatic fibrosis [106]. However, to date, our knowledge regarding the complicated mechanisms responsible for HBV-mediated liver fibrosis remains limited. Although histological evidence has indicated that early liver fibrosis is reversible, and current antiviral treatments can decrease HBV levels to prevent liver fibrosis [7], no available approaches have completely halted the pathological process thus far [8]. Most cases of liver fibrosis progress to cirrhosis. Cirrhosis is irreversible and can lead to hepatic failure and cancer. Because the central event in hepatic fibrosis is HSC activation [107], understanding how HBV activates HSCs can help in designing effective anti-HSC therapeutic strategies to prevent and reverse virus-induced liver fibrosis.

Our reviewed studies have shown that HBV directly stimulates HSC activation [11]. However, whether HBV infects and replicates in HSCs remains controversial. To date, the modulation of HSC activation by HBV-related hepatocytes in a paracrine manner has been well demonstrated. Various soluble inflammatory modulators, including TGF-β [13], CTGF [12], HMGB1 [56], and exosomes [70], can be produced and secreted from HBV-positive liver cells to participate in HSC activation. Among the viral molecules encoded by the virus, the significance of the multifunctional protein HBX in the development of hepatic fibrosis by modulating TGF-β [108], CTGF [50], HMGB1 [58], Wnt5α [66], and exosomes in hepatocytes has been revealed [70]. However, the majority of current evidence on the modulation of HSC mediated by HBX comes from in vitro assays. Further investigations involving animal experiments and clinical samples are required to assess the exact mechanisms underlying HSC activation regulated by the viral molecule. In addition, the immune microenvironment plays a crucial role in the progression of hepatic fibrosis mediated by HBV, and several types of inflammatory cells, including monocytes [76], macrophages [79], Th17 cells [93], NK cells [99], and NKT cells [74], participate in regulating hepatic fibrosis mediated by the virus. However, research focusing on inflammatory cells regulated by the virus is still in its infancy, and the exact mechanisms responsible for the modulation of various HBV-induced inflammatory cells to regulate HSC activation are not well defined. In addition, therapies targeting HSCs to treat HBV-related fibrosis still face significant challenges. Further studies with a focus on HBV-related liver fibrosis are warranted to explore opportunities for curing this disorder.

## Data Availability

Not applicable.

## Abbreviations

HBV: Hepatitis B virus
ECM: Extracellular matrix
TLRs: Toll-like receptors
TRIM37: Tripartite motif-containing protein 37
HSC: Hepatic stellate cells
miRNAs: MicroRNAs
BAMBI: Activin membrane-bound inhibitor
cccDNA: Covalently closed circular deoxyribonucleic acid
IL-6: Interleukin-6
DAMP: Pathogen-associated molecular pattern molecule
CHB: Chronic hepatitis B
